# Pericardial Fluid Biomarkers as Early Predictors for Postoperative Atrial Fibrillation—A Systematic Review

**DOI:** 10.3390/diagnostics15040408

**Published:** 2025-02-07

**Authors:** Florin Mitu, Cristina Andreea Adam, Patricia Richter, Alexandru-Dan Costache, Radu Sebastian Gavril, Clementina Cojocaru, Andrei Țăruș, Mihail Enache, Carmen Marinela Cumpăt, Maria Magdalena Leon, Grigore Tinică

**Affiliations:** 1 Department of Medical and Surgical Specialties I, II and III, “Grigore T. Popa” University of Medicine and Pharmacy, University Street No. 16, 700115 Iasi, Romaniaadcostache@yahoo.com (A.-D.C.);; 2 Cardiovascular Rehabilitation Clinic, Clinical Rehabilitation Hospital, Pantelimon Halipa Street No. 14, 700661 Iasi, Romania; 3 Academy of Medical Sciences, 030167 Bucharest, Romania; 4 Academy of Romanian Scientists, 700050 Iasi, Romania; 5 Rheumatology Clinic, Clinical Rehabilitation Hospital, Pantelimon Halipa Street No. 14, 700661 Iasi, Romania; 6 Cardiovascular Surgery Clinic, “Prof. Dr. George I. M. Georgescu” Cardiovascular Diseases Institute, 700503 Iasi, Romania

**Keywords:** postoperative atrial fibrillation, pericardial fluid, brain natriuretic peptide, interleukin-6, myeloperoxidase, atrial natriuretic peptide, mitochondrial DNA, metabolomics

## Abstract

(1) **Background**: Postoperative atrial fibrillation (POAF) is one of the most common complications of cardiac surgery, frequently occurring in the first 2–4 days after surgery. With a variable incidence depending on the type of surgery, research in recent years has focused on identifying predisposing factors with the aim of correcting them and thus decreasing the risk of cardiovascular and total morbidity and mortality. The analysis of pericardial fluid allowed the identification of biomarkers (interleukin-6, mitochondrial DNA, myeloperoxidase or natriuretic peptides) whose presence postoperatively was associated with increased risk of POAF. (2) **Materials and Methods**: We conducted a search on EMBASE and PubMed and identified 75 articles, of which 10 entered the final analysis. (3) **Results**: Patients who develop POAF accumulate large amounts of interleukin 6, mitochondrial DNA, myeloperoxidase, or secondary atrial natriuretic peptide as a consequence of the associated inflammatory status, atrial remodeling, or disturbance of homeostasis of various ions. There are also observations that their levels in the pericardium correlate with blood levels, but further studies on larger cohorts of patients are needed to provide new evidence in this regard. (4) **Conclusions**: Early recognition of patients at risk of developing POAF based on easy-to-dose and easy-to-use biochemical biomarkers, whose association with POAF has been demonstrated so far in small cohorts of patients, has both therapeutic and prognostic implications, which justifies further research on large cohorts of patients.

## 1. Introduction

Cardiovascular disease is one of the leading causes of death globally, still responsible for a high rate of morbidity despite the prevention strategies implemented. Of the cardiovascular diseases, ischemic coronary heart disease has the highest impact on the age-standardized disability-adjusted life years (DALYs) and the highest mortality rate [1,2]. Since 1816, when the link between the presence of coronary calcifications and angina pectoris was first autopsically demonstrated, and until today, a significant percentage of patients have undergone surgical myocardial revascularization. A decade later, in 1912, Alexis Carrel was awarded the Nobel Prize for his research in the field of arterial anastomosis, which marked the beginning of a new phase in cardiovascular surgery that gradually developed until the 1960s when revascularization techniques began to be widely implemented [3,4,5].

Approximately 85% of patients develop pericardial fluid after cardiac surgery, being frequently associated clinically with the occurrence of right or left heart failure, anterior chest pain, and pericardial friction rub and paraclinically with leukocytosis [6]. In the majority of cases, its presence is transient, with subsequent reabsorption, and anti-inflammatory agents are necessary in a limited number of patients.

Recent studies of postoperative pericardial fluid (PF) has revealed various diagnostic, therapeutic, and prognostic implications by measuring different biomarkers with a role in preventing postoperative atrial fibrillation (POAF) and ventricular dysfunction [7].

**Figure 1:**
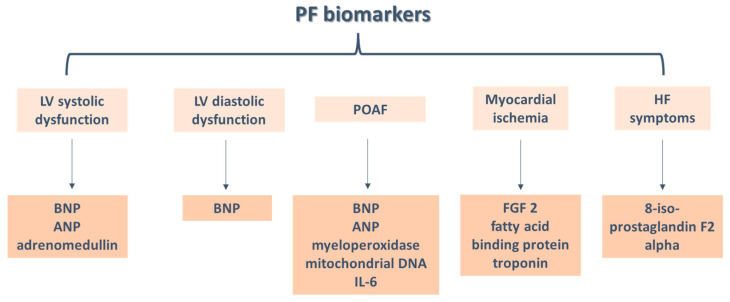
PF biomarkers predicting POAF and ventricular dysfunction (PF: pericardial fluid; LV: left ventricle; BNP: brain natriuretic peptide; ANP: atrial natriuretic peptide; POAF: postoperative atrial fibrillation; IL: interleukin; FGF2: fibroblast growth factor 2).

Thus, high levels of brain natriuretic peptide (BNP) and atrial natriuretic peptide (ANP) correlate with the presence of left ventricular (LV) systolic and diastolic dysfunction, while fibroblast growth factor 2 (FGF 2) correlates positively with the extent of collateral circulation assessed by coronary angiography and with the risk of unstable angina pectoris. The 8-iso-prostaglandin F2alpha level has demonstrated its clinical value by the directly proportional relationship evidenced between these and the severity of HF symptoms or the degree of LV dilatation, while adrenomedullin correlates with LV ejection fraction (LVEF) and end-systolic and end-diastolic volumes of the LV. Markers for myocardial ischemia secondary to cardiac surgery were also identified, with increased fatty acid binding protein and troponin levels being markers correlated with the severity of acute coronary events (Figure 1).

The present study aims to highlight the latest pathophysiologic mechanisms involved in the occurrence of POAF as well as to draw attention to cardiac biomarkers at the PF level that play a role in stratifying patients at risk of developing arrhythmias after cardiac surgery.

## 2. Materials and Methods

### 2.1. Electronic Search Algorithm

We conducted an electronic search in PubMed and EMBASE databases from the time of their inception to January 2025 using different keywords presented in Table 1. This review was performed in accordance with the PRISMA (Preferred Reporting Items for Systematic Reviews and Meta-Analyses) guidelines. Further information on the systematic review protocol is available at the end of the manuscript. The search terms used included various associations between, “pericardial fluid”, “postoperative atrial fibrillation,” and various biomarkers (identified in a *state-of-the-art* review article) including “interleukin-6”, “mitochondrial DNA”, “myeloperoxidase”, “metabolomics”, “brain natriuretic peptide”, and “atrial natriuretic peptide” (one at the time). We used the term pericardial fluid in accordance with similar publications, taking into account the fact that, in a significant percentage of the studies, the analysis is performed from the fluid obtained from the drainage tube, taking into account the fact that sometimes pericardial fluid can be associated with sternal bleeding or small bone fragments. The search retrieved different results for each biomarker, with a total of 81 articles that were evaluated.

### 2.2. Study Selection

A thorough step-by-step analysis of the articles was carried out by two independent reviewers in duplicate and disagreements were resolved by a third independent reviewer. The filters used in the selection process were English language and patient age over 18 years. The studies were organized in the Mendeley reference manager (https://www.mendeley.com/ accessed on 1 August 2024) and subsequently registered in a spreadsheet for data extraction and organization. We aimed to include all the reports that include details about the following characteristics: the study design, type of cardiac surgery, number of patients, mean age, follow-up period, outcome (occurrence of POAF after cardiac surgery) and data regarding the pericardial level of biomarkers in patients with POAF.

**Figure 2:**
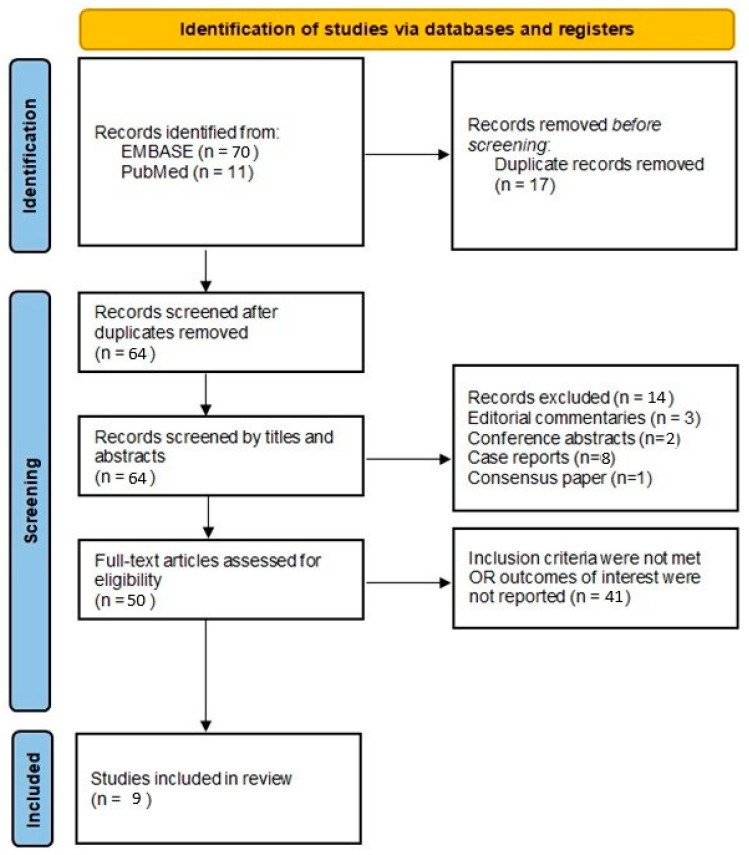
Flow chart of selected studies in the present analysis.

After removing duplicates, a total of 64 articles were analyzed by title and abstract. The title and the abstract were the first aspects analyzed by the 2 reviewers. In the case of abstracts, the articles were selected in which the diagnosis of POAF was mentioned and referred to biomarkers dosed in the pericardial fluid in relation to its occurrence. Of these, 11 were excluded on the basis of article type as follows: 3 editorial comments, 2 abstracts published at a conference, 8 clinical cases, and 1 review article; 41 of the articles did not meet the inclusion and exclusion criteria, so the final number of articles evaluated was 9 (Figure 2). Also, 2 of the studies reported on the status of the pericardium at the end of surgery, in both of which it was re-approximated [8,9].

### 2.3. Quality Assessment

**Figure 3:**
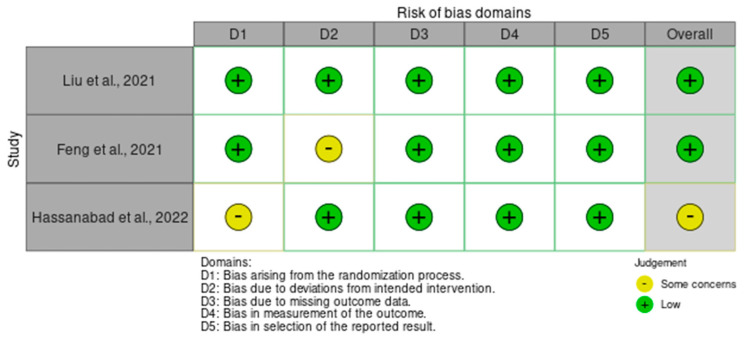
Authors’ assessments of each methodological quality item for every study that was included in the methodological quality summary—IL6 ([8,9,10]).

**Figure 4:**
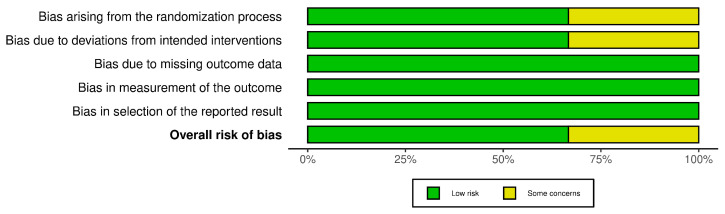
Different kinds of biases (domains) in the studies that were part of the systematic review for IL-6.

**Figure 5:**
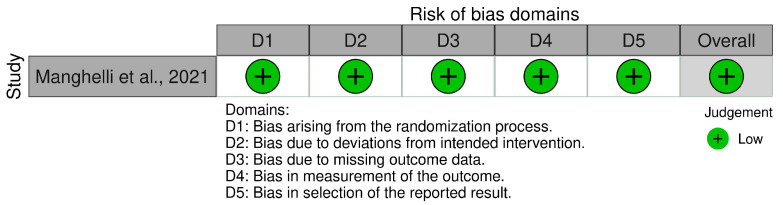
Authors’ assessments of each methodological quality item for every study that was included in the methodological quality summary—mitochondrial DNA [11].

**Figure 6:**
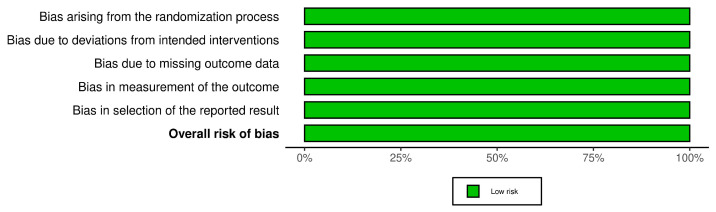
Different kinds of biases (domains) in the studies that were part of the systematic review for mitochondrial DNA.

**Figure 7:**
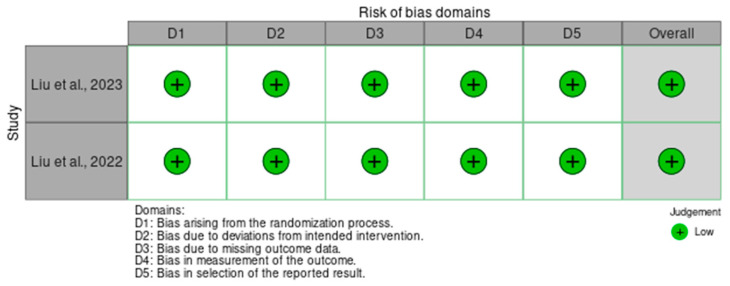
Authors’ assessments of each methodological quality item for every study that was included in the methodological quality summary—myeloperoxidase [12,13].

**Figure 8:**
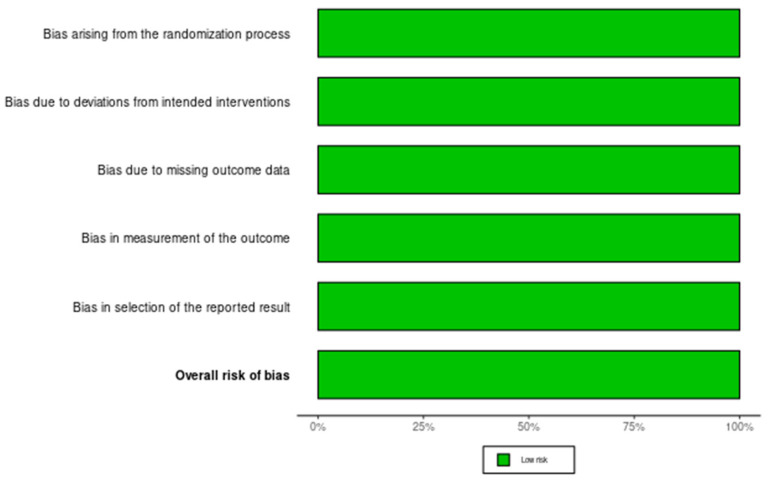
Different kinds of biases (domains) in the studies that were part of the systematic review for X myeloperoxidase.

In order to assess the quality of the studies included in our systematic review, we have performed a quality check using the risk of bias assessment tool. The results can be observed in Figure 3 and Figure 4 for interleukin (IL) 6, Figure 5 and Figure 6 for mitochondrial DNA, respectively, and Figure 7 and Figure 8 for myeloperoxidase.

We have analyzed five domains in evaluating the risk of bias: bias arising from the randomization process, bias due to deviations from the intended intervention, bias due to missing outcome data, bias in measuring the outcome, and bias in selecting the reported result. None of the articles included in our systematic review had a high risk of bias, while some concerns were identified in 25% for the overall risk of bias.

## 3. Results

Several characteristics of the included studies were detailed—sample size, age at surgery moment, type of surgery, outcome, follow-up period, and results—as can be seen in Table 2. From an epidemiologic point of view, patients of different ethnicities from various geographic areas were included.

Two of the studies that evaluated the role of IL-6 dosing in FP in the occurrence of POAF predominantly included patients undergoing CABG [8,9,10]. In all of the studies presented in Table 2, IL-6 was shown to be an independent predictor, with the associated relative risk being 2–3 times higher. Based on data from multivariate statistical analysis, Feng et al. [8] developed a prediction model of POAF based on six clinical, biological, and echocardiographic parameters in which the identification of an IL6 level in pericardial fluid above 66 ng/mL at 12 h postoperatively is associated with a 3.19-fold increased relative risk of POAF (*p* = 0.004).

One clinical trial [11] evaluated the role of mitochondrial DNA in the development of POAF, led by the same team of investigators. The patients included in the study underwent various surgical procedures (CABG, valve prosthesis), and the follow-up period of mitochondrial DNA levels was up to 48 h postoperatively. The subgroups of patients who developed POAF showed higher values of the incriminated biomarker, but further research involving different investigators, different populations, different surgeries, and different interventions is needed to provide further evidence in this regard. The presence of mitochondrial DNA at 24 h was associated in multivariate statistical analysis with a relative risk of 1.071 for POAF (*p* = 0.007).

Along with IL-6 and mitochondrial DNA, the presence of high titers of pericardial myeloperoxidase correlates with the occurrence of POAF, with the relative risk reported by Liu et al. [12] being 19 times higher than in surgical patients who do not develop POAF. Myeloperoxidase dosing after CABG improved the diagnostic power of a clinically defined parameter-based model by increasing the area under the curve from 0.760 to 0.901. In another clinical study, Liu et al. [13] demonstrated that the presence of a myeloperoxidase level higher than 17,735.50 ng/mL in pericardial fluid has a sensitivity of 96% and a specificity of 56.50% to predict the occurrence of POAF (area under the curve: 0.805). A single clinical trial [14] has analyzed the role of metabolomes as predictors of POAF with promising results, but further research in larger groups of patients is needed. This group of investigators demonstrated that the pericardial fluid levels of arginine, methionine, ornithine, and aceglutamide are higher in patients with POAF (*p* < 0.001 for all parameters), and the model generated on its association was found to have high statistical significance (area under the curve: 0.96; 95% CI: 0.929–0.988).

Nakamura et al. [15] demonstrated in a study including 42 patients undergoing CABG that patients who developed POAF had serum pericardial BNP levels approximately 5-fold higher, with each 50 pg/mL increase in serum BNP associated with a tripling of risk (*p* = 0.04, 95% CI 1.1–8.6). NT-proBNP and GDF-15 are two biomarkers whose presence in pericardial fluid was associated with the occurrence of POAF (area under the curve: 0.85 for NT-proBNP and 0.68 for GDF-15 [16]).

**Table 2:** Main studies reporting predicting value of PF biomarkers in POAF occurrence.

Author, Year	Design	Patiens, No	Age (Mean/Median ± SD)	Type of Cardiac Surgery	Outcome	Follow-Up Period	Results
Liu et al., 2021 [9]	Observational, multicenter	124	65.91 ± 8.27 (POAF group) 58.26 ± 9.22 (non-POAF group)	CABG	POAF	18 h postop	Pericardial IL-6 is associated with a 2.154× higher risk of developing POAF.
Feng et al., 2021 [8]	Prospective cohort study	227	63.83 ± 8.79	CABG	POAF	12 h postop	Pericardial IL-6 ≥ 166 ng/mL is associated with a 3.19× higher risk of developing POAF.
Hassanabad et al., 2022 [10]	Prospective cohort study	43	62.4 ± 12.9	CABG AVR CABG + AVR Minimally invasive cardiac surgery	Characterization of the immunological profile and role in the development of POAF	up to 48 h postop	IL-6, MMP-9, and TIMPs had increased postop pericardial values.
Manghelli et al., 2021 [11]	Prospective cohort study	100	65 ± 9 (POAF group) 61 ± 10 (non-POAF group)	CABG Heart valve surgery Both	POAF	4, 12, 24, 48 h postop	Pericardic mitochondrial DNA levels at 24 h postop are predictors for POAF.
Liu et al., 2023 [12]	Prospective cohort study	137	N/A	Isolated CABG	POAF	6, 12, 18 h postop	Pericardial myeloperoxidase levels at 6 h postop are predictors for POAF (OR 19.215).
Liu et al., 2022 [13]	Prospective cohort study	97	62–64 years old	CABG	POAF	Until discharge	Pericardial MPO levels above 17,735 ng/mL are independent predictors for POAF (sensitivity 96.20%, specificity 56.50%).
Yang et al., 2023 [14]	Prospective cohort study	50	N/A	CABG	POAF POAF-associated metabolic alterations	6, 12, 18 h postop	Aceglutamide, ornithine, methionine, and arginine are predictors for POAF.
Fragão-Marques et al., 2021 [16]	Prospective cohort study	65 PF, 95 serum and 36 atrial samples	72.2 ± 8.4	aortic valve replacement surgery	MACE (stroke, acute myocardial infarction, all-cause mortality and major arrhythmia–ventricular fibrillation, third-grade atrioventricular block); acute kidney injury POAF	48 h	PF GDF-15 was similar regardless of POAF occurrence. PF NT-pro-BNP had similar values independent of MACE, POAF occurrence, bleeding or hospital stay.
Nakamura et al., 2007 [15]	Prospective cohort study	42	71 ± 6 (POAF group) 63 ± 9 (non-POAF group)	CABG	POAF	Until discharge	PF BNP was higher in the POAF group. PF BNP is accompanied by a three-fold risk of developing POAF with every 50 pg/mL increase in level. No significant difference in PF ANP between groups.

SD: standard deviation; CABG: coronary artery bypass grafting; PF: pericardial fluid; POAF: postoperative atrial fibrillation; AVR: aortic valve replacement; MMP-9: matrix metalloproteinase 9; TIMPs: tissue inhibitors of matrix metalloproteinase; MPO: myeloperoxidase; N/A: not available; GDF-15: growth/differentiation factor 15; NT-pro-BNP: N-terminal pro b-type natriuretic peptide; IL: interleukin; BNP: brain natriuretic peptide; ANP: atrial natriuretic peptide;

## 4. Discussion

POAF is the most common complication after heart surgery [17] and is associated with a high risk of mortality and acute cardiovascular events (such as stroke) or heart failure [18]. The pathophysiologic mechanisms are not clearly understood. Having been extensively investigated in recent years in view of its increasing incidence, several pathophysiologic mechanisms have been incriminated, including changes in atrial structure, the presence of PF in association with inflammation, metabolic activity of adipose tissue surrounding the atria [19], myocardial ischemia, alterations in ion channels, or re-entry mechanisms in pulmonary veins [20,21,22,23].

### 4.1. Pericardial Fluid After Cardiac Surgery—Occurrence and Importance of Biochemical Analysis

The occurrence of PF after cardiac surgery is common, with the incidence increasing due to increasingly complex surgeries or the use of chronic anticoagulation [24,25]. Longer hospitalization and increased risk of mortality in the context of cardiac tamponade or the occurrence of arrhythmias are some of the arguments that support the importance of understanding the pathophysiological mechanisms associated with its composition [26].

**Figure 9:**
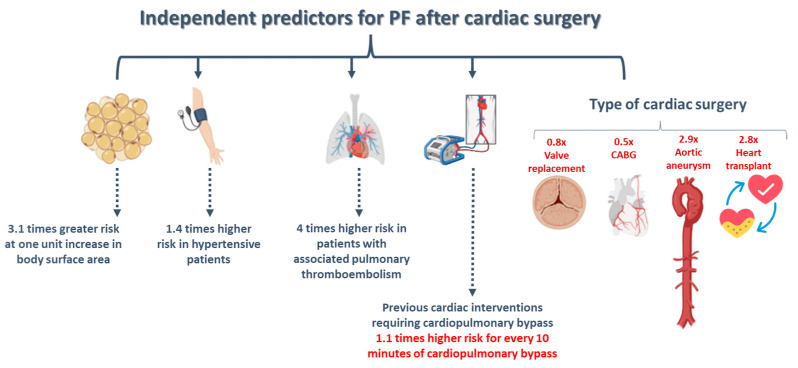
Predictors for PF after cardiac surgery (PF: pericardial fluid; CABG: coronary artery bypass grafting) (illustration created with Biorender software version 04).

Ashikhmina et al. [27] conducted a retrospective clinical study on a cohort of 21,416 patients who underwent various cardiac surgeries with cardiopulmonary bypass between 1993 and 2005. The investigators demonstrated a prevalence of PF of only 1.5, with over 85% of patients being without specific symptoms. By comparing the group of patients with and without PF, a number of independent predictors for postoperative occurrence of PF were identified, as can be seen in Figure 9.

The observation that the incidence of PF varies according to the type of surgery has been confirmed by other similar studies reported so far in the literature. Thus, in patients with coronary artery bypass grafting (CABG), the rate of PF was lower due to the involvement of the pleural space by internal mammary artery sampling. In the case of aortic interventions, those at the aortic root level are accompanied by the highest risk of pleural fluid, the explanation being the higher rate of early postoperative bleeding, which leads to the formation of mediastinal blood clots that stimulate local inflammatory processes. Also, the lysis of this blood clot accentuates the accumulation of fluid by osmosis around the perigraft space. Another potential mechanism is excessive bleeding, which has a higher chance of association with a remaining thrombus or extensive mediastinal resection with lymphatic circulation involvement.

Heart transplantation is accompanied by a number of factors that predispose patients to fluid accumulation in the pericardium. Thus, dilated cardiomyopathy, immunosuppressive treatment with cyclosporine, acute graft rejection, or differences in anthropometric parameters between donor and recipient may cause the occurrence of PF [27]. The concomitant association of rheumatologic pathologies (rheumatoid polyarthritis, systemic lupus erythematosus) that require the administration of immunosuppressive medication preoperatively (corticosteroids and cytostatics) increases the risk of coagulopathies or immune disorders after heart transplantation.

A general clinical observation is the frequent association of PF with pleural fluid, especially bilateral pleural fluid secondary to heart failure or inflammatory reaction secondary to the stressor associated with surgery and, to a lesser extent, postoperative bleeding [28]. Similar clinical research has shown that prolonged donor ischemic time also predisposes patients to fluid accumulation in the pericardial space [29]. Renal dysfunction and a prolonged cardiopulmonary bypass time are other parameters associated with the occurrence of PF, with the pathophysiologic hypothesis being the associated systemic inflammatory response [27].

### 4.2. Postpericardiotomy Syndrome

Postpericardiotomy syndrome has an incidence of up to 30% in patients undergoing cardiac surgery as a result of an autoimmune inflammatory reaction involving the pleural and pericardial spaces [30]. According to clinical research conducted by Gabaldo et al., the incidence varies depending on the type of surgery, most commonly associated with aortic valve prosthesis or aortic pathology (20–30%), while in CABG or mitral valve replacement, the percentages are much lower (up to 10% of cases) [31]. Among the subgroup of patients undergoing aortic valve surgery, a number of predictors were identified, such as age, urgency of surgery, postoperative fever, elevated serum C reactive protein values above 5 mg/L, or antibiotic therapy (*p* < 0.05 for all parameters) [31].

Female gender, perioperative incision of the pleura, blood transfusions, or patients with diabetes mellitus who are not on metformin therapy are other clinical–paraclinical elements that increase the risk of postpericardiotomy syndrome [32,33].

Several clinical studies highlight increased body mass index as a protective factor through the immunomodulatory effect of obesity secondary to increased serum levels of interleukins with an anti-inflammatory role (IL-4, IL-13) [33,34]. Another aspect intensively analyzed in relation to postpericardiotomy syndrome is the occlusion of grafts used in aortocoronary bypass [28] secondary to hyperemic myxedematous hyperemic inflammation that causes dense areas of obliterative fibrosis. Data in the literature are inconclusive, and more extensive research on large groups of patients is needed to prove or disprove the hypothesis [35]. Ikäheimo et al. [28] analyzed a group of 150 patients undergoing cardiac surgery (50-valve replacement, 100-CABG) and found the presence of PF in 77% of them. The group of investigators observed the frequent association of atrial arrhythmias, cardiac dilatation, or pleural fluid in the group of patients with PF. Also, the usage of the left internal mammary artery (LIMA) as a graft for patients undergoing CABG was associated with an increased incidence of PF (*p* < 0.005)—through associated mediastinal bleeding. This hypothesis has recently been disproved by a group of Serbian researchers who analyzed a group of more than 1900 patients (predominantly male, average age of 57 years) who underwent CABG. On the basis of the type of graft used, two subgroups of patients were organized: 1468 patients who used an arterial graft (LIMA) and a secondary group of 461 patients who used a venous graft. FP was targeted in approximately equal percentages in the two groups (63.4% vs. 62.4%), but the localization of the accumulation was associated with statistical significance; in 41.3% of all cases, the disposition was circular (*p* < 0.001) [36]. A much less traumatic alternative with a faster recovery and a lower complication rate is the use of both internal mammary arteries for BACG and the use of the da Vinci robot [37], which currently provides the most durable results. The method is associated with a lower risk of stroke by limiting aortic manipulation, avoiding sternotomy, and thus reducing the risk of developing PF [38,39,40].

### 4.3. Biochemical and Molecular Composition of PF

**Figure 10:**
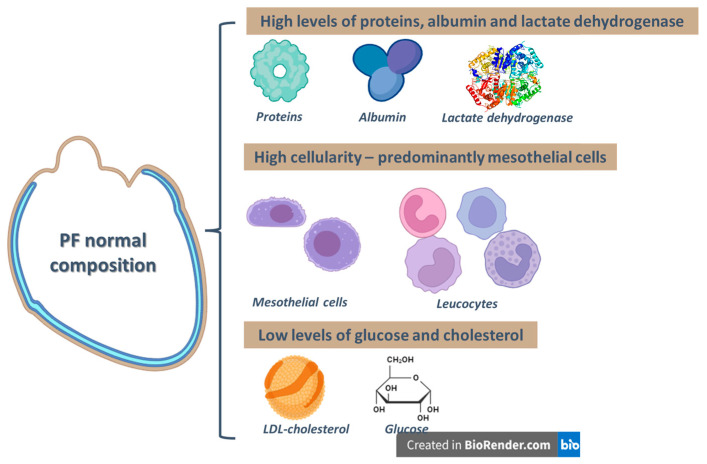
PF normal composition (PF: pericardial fluid; LDL: low-density lipoprotein) (illustration created with Biorender software version 04) (adapted after [41]).

The PF analysis revealed a high content of nucleated cells and proteins of various molecular sizes (Figure 10) originating from the myocardial interstitium by crossing the epicardium. The trauma generated by the surgical intervention causes the initial production and subsequent release of various inflammatory mediators that, through venous drainage or interstitial diffusion, reach the pericardium and can be quantified in the blood circulation [7,41,42].

A maximum of 50 mL of serous fluid is normally found in the pericardial sac [43,44]. The differentiation between transudate and exudate using the Light criteria [45] is debated in the literature, with some clinical investigations being misclassified. Buoro et al. [41] analyzed FP biochemically and cytologically in a group of 120 patients and found elevated levels of protein, albumin, and lactate dehydrogenase along with a high cellularity mainly comprising mesothelial cells. The mean glucose content is 80–134 mg/dL, total cholesterol is between 12 and 70 mg/dL, and LDH is between 141 and 2613 U/L. Optical microscopy revealed the presence of leukocytes (with a high percentage of lymphocytes [46]) together with mesothelial cells [47]. Imazio et al. [48] analyzed the FP composition in a group of 50 patients who underwent cardiac surgery and found a 50% lower value for pericardial sac versus blood proteins and a unity ratio for lactate dehydrogenase.

Bai et al. [49] developed a predictive model to identify transudates based on the observation that Light criteria misclassify PF as exudate in up to 30% of patients, especially in those on diuretic therapy. The parameters included in the model were a protein gradient greater than 23 g/L, pleural fluid lactate dehydrogenase level, its ratio to the lactate dehydrogenase serum level, pleural fluid adenosine deaminase level, and N-terminal fragment of pro-brain natriuretic peptide (NT-proBNP) value. These predictors have the ability to identify pericardial collections with a very high accuracy rate (area under the curve of 0.953). Also, the presence of exudates is statistically more likely in patients over 75 years of age who have a protein gradient of more than 31 g/L.

### 4.4. PF Biomarkers for POAF

POAF increases the risk of recurrence of atrial fibrillation in the long term (3.52 times higher relative risk after adjusting for different cofounders such as age, gender, or clinical and surgical factors) [50], a risk that also depends on the type of surgery performed; for valve replacement, the risk is up to 50%, while in CABG the percentage decreases to 20%, and in heart transplantation it is up to 5%. Not only cardiac surgery is a risk factor for POAF; about 30% of patients undergoing pneumectomy develop this arrhythmia.

The main triggers of POAF in the postoperative period are oxidative stress, inflammation, activation of the autonomic nervous system, and blood accumulation in the pericardium, which justifies the need to identify easily determinable, feasible, reproducible biomarkers that contribute to risk stratification of developing POAF [51]. The occurrence of POAF is also determined by myocardial reperfusion lesions, which at the atrial level causes a reduction in mitochondrial processes at the myocyte level, thus stimulating the arrhythmogenic process by loss of calcium homeostasis and the production of reactive oxygen species (ROS) [51,52].

In a meta-analysis that included a total of 19 randomized clinical trials enrolling approximately 3425 patients, a group of researchers demonstrated that posterior pericardial drainage is a simple and safe therapeutic option that reduces the risk of POAF by approximately 60% (*p* < 0.001) and therefore statistically significantly reduces the risk of death or cardiac tamponade (*p* = 0.03) [53]. Similar results have been reported in several clinical studies in the case of left posterior pericardiotomy, with the rate of occurrence of PE in patients who underwent pericardiotomy being significantly lower (12% vs. 21%) [54,55].

#### 4.4.1. Interleukin-6

IL-6 is a biomarker with a pro-inflammatory role that increases the rate of POAF by mediating signaling pathways regulated by transcriptase-3 activation and stimulating cardiac fibroblast proliferation [7]. Local trauma secondary to CABG causes the development of an inflammatory status that subsequently stimulates the synthesis and release in the pericardial cavity of several cytokines, including IL-6 [56]. Liu et al. [9] demonstrated in a group of 124 patients undergoing CABG the biomarker value of pericardial IL-6 in the occurrence of POAF 18 h after surgery; 35% of the patients developed POAF within the first 2 days postoperatively, with these patients having a clinical picture dominated by advanced age, the presence of cerebrovascular disease, extensive surgical drainage, and elevated pericardial IL-6 levels. Multivariate statistical analysis revealed the volume of drained FP, the presence of hypertension, and cerebrovascular disease or the use of angiotensin-converting enzyme inhibitors or sartans as predictors of POAF in addition to IL-6 and age. In the POAF group, the pericardial IL-6 level was 26 times higher compared to the blood value and strengthened the argument that it can be used as a biomarker in the occurrence of arrhythmia.

In a similar clinical trial, Feng et al. [8] developed a prediction model for POAF using clinical, biological, and echocardiographic parameters: age ≥ 61 years (2 points), left atrium diameter ≥ 49 mm and right atrium diameter ≥ 45 mm (1 point each), more than 3 grafts used (1 point), serum uric acid level ≥ 226 µmol/L 12 h postoperatively (1 point), and pericardial IL-6 level ≥ 166 ng/L 12 h postoperatively (2 points).

#### 4.4.2. Metalloproteinases

The increased activity of matrix metalloproteinases stimulates the process of scar remodeling in the myocardium, thus outlining the heterogeneity of electrical conduction and atrial refractivity that will stimulate the occurrence of POAF [57].

Hassanabad et al. [10] demonstrated increased concentrations of both metalloproteinases (especially MMP-9) and their tissue inhibitors (TIMPs) in the pericardial cavity following cardiac surgery. The inflammatory status associated with the presence of these cytokines modulates various pathophysiologic processes involved in the development of POAF and postpericardiotomy syndrome. MMP evolution in the pericardial cavity is different depending on the molecules. Thus, the same group of investigators demonstrated that, postoperatively, the pericardial MMP-8 level increased significantly, while the MMP-9 concentration decreased, a decrease that can be explained by the lack of postoperative macrophages (which are the main cells responsible for regulating MMP-9 expression) [58,59,60].

#### 4.4.3. Mitochondrial Deoxyribonucleic Acid

Mitochondria mediate the pathophysiological processes involved in the development of POAF by generating ROS. Mitochondrial DNA is released by cardiomyocytes in the context of hemodynamic stress, direct trauma, or ischemia [61]. The presence of mitochondrial DNA in the pericardial cavity contributes to the promotion of the pro-inflammatory status that predisposes patients to POAF [11].

Sandler et al. [62] analyzed a group of 16 patients who underwent various cardiac surgeries (11 patients—CABG; 5—aortic or mitral valve replacement with/without CABG) and demonstrated a significant increase in mitochondrial DNA after surgery—6-fold higher immediately postoperatively and 5-fold higher after 24–48 h (*p* = 0.02). Postoperatively, increases in mitochondrial DNA were recorded irrespective of the presence or absence of POAF, but increases in this level of at least 2× predisposed patients to POAF (37.5% of all cases) (*p* = 0.037).

#### 4.4.4. Metabolomics

Analysis of metabolomes in relation to POAF raises the hypothesis of new pathophysiologic mechanisms that both explain the occurrence of arrhythmia and indirectly identify patients at risk [14,63]. Dosing metabolites with molecular weights less than 1500 Da is a technological advancement in precision, individualized therapy. Metabolomics changes reflect changes in the genome, transcriptome, proteome level on a macroscopic scale [64,65,66,67,68].

Recent research has identified over 60 metabolites in the plasma of a group of POAF patients [69]. The urea cycle, betaine metabolism, and various amino acid metabolism pathways were implicated in relation to the metabolic pathways that contributed to the development of POAF [14].

#### 4.4.5. Myeloperoxidase 

Myeloperoxidase (MPO) is a leukocyte-derived inflammatory enzyme (known also as a promising biomarker) that maintains the pro-inflammatory status in the pericardium by producing ROS. The link between MPO and AF has been previously demonstrated, with elevated levels found in patients with paroxysmal AF, and increases the risk of arrhythmia recurrence after ablation [70,71]. In addition to its pro-arrhythmogenic role, MPO also modulates atherosclerotic processes by initiating lipid peroxidation and accelerating post-translational changes in various proteins that modulate diverse pathophysiologic processes [72,73]. MPO has also been implicated in atrial remodeling, which supports its role as a pericardial biomarker with a stratifying role in the risk of POAF. Myeloperoxidase has also been incriminated as having an active role in the progression of atherosclerotic processes [12,70].

#### 4.4.6. Natriuretic Peptides

Comparative analysis of the predictive value of the pericardial value of NT-proBNP versus that of the blood showed that the pericardial biomarker assay provides results with superior accuracy, correlating with the degree of atrial remodeling, thus being effective for predicting POAF in patients undergoing surgery and their stratification [16]. Michaud et al. [74] analyzed postmortem a total of 96 patients and measured NT-proBNP levels in FP, demonstrating higher levels in patients with chronic ischemia, with or without previous acute events. They also observed the existence of positive correlations between biomarker levels in blood and FP, which both justifies and encourages research focused on FP analysis.

In the case of both BNP and ANP, their dosing in pericardium is superior to serum analysis. In a group of 42 patients undergoing off-pump CABG, 21% of them developed POAF, on average, 3 days after surgery. In order to profile these patients, the investigators assessed serum peptide levels and demonstrated higher levels of serum peptides in the BNP group of patients with POAF compared to those without POAF (*p* = 0.0001). In ANP, no significant differences were found between the two groups [15].

#### 4.4.7. Clinical Implications and Future Directions

Although it is still at the stage of small-scale research, the demonstration of the existence of biomarkers that are easily determinable by analysis of pericardial fluid justifies the development of multicenter, randomized clinical trials, which will provide additional evidence. The clinical impact is significant by limiting the rate of occurrence of POAF, decreasing both the duration of hospitalization and the prognosis in the short and long term. It is also beneficial to establish validated cut-off values in a variety of patient cohorts to serve as useful clinical tools for cardiologists and surgeons alike. Last but not least, further research on metabolomes and the pathophysiological mechanisms behind them will allow a deeper understanding of the pathophysiological mechanisms involved in the occurrence of POAF.

The present study has a number of limitations, mainly due to the small number of prospective studies on the analysis of postoperative pericardial fluid to identify biomarkers associated with the occurrence of POAF and the variable time interval of POAF reported in the studies included in this systematic review. Also, the use of language filters may result in the exclusion of similar research of clinical and prognostic value.

## 5. Conclusions

IL-6, mitochondrial DNA, myeloproteinases, natriuretic peptides, and some metabolomes are biomarkers whose presence in FP is associated with a high risk of developing POAF. The existing data so far are limited, but the results are promising and emphasize the positive predictive role, thus contributing to a better understanding of the multifactorial origin of POAF.

## Figures and Tables

**Table 1:** Search strategy in EMBASE and PubMed.

Biomarker	Terms	Query	Results
IL-6	pericardial fluid AND postoperative atrial fibrillation AND interleukin-6	**EMBASE** (“pericardial fluid”/exp OR “pericardial fluid” OR (pericardial AND (“fluid”/exp OR fluid))) AND (“postoperative atrial fibrillation”/exp OR “postoperative atrial fibrillation” OR (postoperative AND atrial AND (“fibrillation”/exp OR fibrillation))) AND (“interleukin 6”/exp OR “interleukin 6”) **PubMed** (“pericardial fluid” [MeSH Terms] OR (“pericardial” [All Fields] AND “fluid” [All Fields]) OR “pericardial fluid” [All Fields]) AND ((“postoperative period” [MeSH Terms] OR (“postoperative” [All Fields] AND “period” [All Fields]) OR “postoperative period” [All Fields] OR “postop” [All Fields] OR “postoperative” [All Fields] OR “postoperatively” [All Fields] OR “postoperatives” [All Fields]) AND (“atrial fibrillation” [MeSH Terms] OR (“atrial” [All Fields] AND “fibrillation” [All Fields]) OR “atrial fibrillation” [All Fields])) AND (“interleukin 6” [MeSH Terms] OR “interleukin 6” [All Fields] OR “interleukin 6” [All Fields])	EMBASE—11 results PubMed—2 results
Mitochondrial DNA	pericardial fluid AND postoperative atrial fibrillation AND mitochondrial DNA	**EMBASE** (“pericardial fluid”/exp OR “pericardial fluid” OR (pericardial AND (“fluid”/exp OR fluid))) AND (“postoperative atrial fibrillation”/exp OR “postoperative atrial fibrillation” OR (postoperative AND atrial AND (“fibrillation”/exp OR fibrillation))) AND (“mitochondrial dna”/exp OR “mitochondrial dna”) **PubMed** (“pericardial fluid” [MeSH Terms] OR (“pericardial” [All Fields] AND “fluid” [All Fields]) OR “pericardial fluid” [All Fields]) AND ((“postoperative period” [MeSH Terms] OR (“postoperative” [All Fields] AND “period” [All Fields]) OR “postoperative period” [All Fields] OR “postop” [All Fields] OR “postoperative” [All Fields] OR “postoperatively” [All Fields] OR “postoperatives” [All Fields]) AND (“atrial fibrillation” [MeSH Terms] OR (“atrial” [All Fields] AND “fibrillation” [All Fields]) OR “atrial fibrillation” [All Fields])) AND (“dna, mitochondrial” [MeSH Terms] OR (“dna” [All Fields] AND “mitochondrial” [All Fields]) OR “mitochondrial dna” [All Fields] OR (“mitochondrial” [All Fields] AND “dna” [All Fields]))	EMBASE—4 results PubMed—1 result
Myeloperoxidase	pericardial fluid AND postoperative atrial fibrillation AND myeloperoxidase	**EMBASE** (“pericardial fluid”/exp OR “pericardial fluid” OR (pericardial AND (“fluid”/exp OR fluid))) AND (“postoperative atrial fibrillation”/exp OR “postoperative atrial fibrillation” OR (postoperative AND atrial AND (“fibrillation”/exp OR fibrillation))) AND (“myeloperoxidase”/exp OR “myeloperoxidase”) **PubMed** (“pericardial fluid” [MeSH Terms] OR (“pericardial” [All Fields] AND “fluid” [All Fields]) OR “pericardial fluid” [All Fields]) AND ((“postoperative period” [MeSH Terms] OR (“postoperative” [All Fields] AND “period” [All Fields]) OR “postoperative period” [All Fields] OR “postop” [All Fields] OR “postoperative” [All Fields] OR “postoperatively” [All Fields] OR “postoperatives” [All Fields]) AND “atrail” [All Fields] AND (“fibril” [All Fields] OR “fibril s” [All Fields] OR “fibrilation” [All Fields] OR “fibrilization” [All Fields] OR “fibrilized” [All Fields] OR “fibrillate” [All Fields] OR “fibrillated” [All Fields] OR “fibrillates” [All Fields] OR “fibrillating” [All Fields] OR “fibrillation” [All Fields] OR “fibrillations” [All Fields] OR “fibrillization” [All Fields] OR “fibrillize” [All Fields] OR “fibrillized” [All Fields] OR “fibrillizes” [All Fields] OR “fibrillizing” [All Fields] OR “fibrillous” [All Fields] OR “fibrills” [All Fields] OR “fibrils” [All Fields])) AND (“myeloperoxidases” [All Fields] OR “peroxidase” [MeSH Terms] OR “peroxidase” [All Fields] OR “myeloperoxidase” [All Fields])	EMBASE—5 results PubMed—3 results
Metabolomics	pericardial fluid AND postoperative atrial fibrillation AND metabolomics	**EMBASE** (“pericardial fluid”/exp OR “pericardial fluid” OR (pericardial AND (“fluid”/exp OR fluid))) AND (“postoperative atrial fibrillation”/exp OR “postoperative atrial fibrillation” OR (postoperative AND atrial AND (“fibrillation”/exp OR fibrillation))) AND (“metabolomics”/exp OR “metabolomics”) **PubMed** (“pericardial fluid” [MeSH Terms] OR (“pericardial” [All Fields] AND “fluid” [All Fields]) OR “pericardial fluid” [All Fields]) AND ((“postoperative period” [MeSH Terms] OR (“postoperative” [All Fields] AND “period” [All Fields]) OR “postoperative period” [All Fields] OR “postop” [All Fields] OR “postoperative” [All Fields] OR “postoperatively” [All Fields] OR “postoperatives” [All Fields]) AND (“atrial fibrillation” [MeSH Terms] OR (“atrial” [All Fields] AND “fibrillation” [All Fields]) OR “atrial fibrillation” [All Fields])) AND (“metabolome” [MeSH Terms] OR “metabolome” [All Fields] OR “metabolomes” [All Fields] OR “metabolomics” [MeSH Terms] OR “metabolomics” [All Fields] OR “metabolomic” [All Fields])	EMBASE—2 results PubMed—1 result
BNP	pericardial fluid AND postoperative atrial fibrillation AND brain natriuretic peptide	**EMBASE** (“pericardial fluid”/exp OR “pericardial fluid” OR (pericardial AND (“fluid”/exp OR fluid))) AND (“postoperative atrial fibrillation”/exp OR “postoperative atrial fibrillation” OR (postoperative AND atrial AND (“fibrillation”/exp OR fibrillation))) AND (“brain natriuretic peptide”/exp OR “brain natriuretic peptide”) **PubMed** (“pericardial fluid” [MeSH Terms] OR (“pericardial” [All Fields] AND “fluid” [All Fields]) OR “pericardial fluid” [All Fields]) AND ((“postoperative period” [MeSH Terms] OR (“postoperative” [All Fields] AND “period” [All Fields]) OR “postoperative period” [All Fields] OR “postop” [All Fields] OR “postoperative” [All Fields] OR “postoperatively” [All Fields] OR “postoperatives” [All Fields]) AND (“atrial fibrillation” [MeSH Terms] OR (“atrial” [All Fields] AND “fibrillation” [All Fields]) OR “atrial fibrillation” [All Fields])) AND (“natriuretic peptide, brain” [MeSH Terms] OR (“natriuretic” [All Fields] AND “peptide” [All Fields] AND “brain” [All Fields]) OR “brain natriuretic peptide” [All Fields] OR (“brain” [All Fields] AND “natriuretic” [All Fields] AND “peptide” [All Fields]))	EMBASE—38 results PubMed—2 results
ANP	pericardial fluid AND postoperative atrial fibrillation AND atrial natriuretic peptide	**EMBASE** (“pericardial fluid”/exp OR “pericardial fluid” OR (pericardial AND (“fluid”/exp OR fluid))) AND (“postoperative atrial fibrillation”/exp OR “postoperative atrial fibrillation” OR (postoperative AND atrial AND (“fibrillation”/exp OR fibrillation))) AND (“atrial natriuretic peptide”/exp OR “atrial natriuretic peptide”) **PubMed** (“pericardial fluid” [MeSH Terms] OR (“pericardial” [All Fields] AND “fluid” [All Fields]) OR “pericardial fluid” [All Fields]) AND ((“postoperative period” [MeSH Terms] OR (“postoperative” [All Fields] AND “period” [All Fields]) OR “postoperative period” [All Fields] OR “postop” [All Fields] OR “postoperative” [All Fields] OR “postoperatively” [All Fields] OR “postoperatives” [All Fields]) AND (“atrial fibrillation” [MeSH Terms] OR (“atrial” [All Fields] AND “fibrillation” [All Fields]) OR “atrial fibrillation” [All Fields])) AND (“atrial natriuretic factor” [MeSH Terms] OR (“atrial” [All Fields] AND “natriuretic” [All Fields] AND “factor” [All Fields]) OR “atrial natriuretic factor” [All Fields] OR (“atrial” [All Fields] AND “natriuretic” [All Fields] AND “peptide” [All Fields]) OR “atrial natriuretic peptide” [All Fields])	EMBASE—10 results PubMed—2 results

IL: interleukin; BNP: brain natriuretic peptide; ANP: atrial natriuretic peptide.

## Data Availability

The study protocol can be found at dx.doi.org/10.17504/protocols.io.x54v92be1l3e/v1 accessed on 1 August 2024. We also mention that this systematic review was conducted in accordance with PRISMA guidelines.

## References

[1] Vaduganathan M., Mensah G.A., Turco J.V., Fuster V., Roth G.A. (2022). The Global Burden of Cardiovascular Diseases and Risk. J. Am. Coll. Cardiol..

[2] Roth G.A., Mensah G.A., Fuster V. (2020). The Global Burden of Cardiovascular Diseases and Risks. J. Am. Coll. Cardiol..

[3] Tully A., Bishop M.A. (2024). Coronary Artery Surgery. StatPearls.

[4] Greason K.L., Schaff H.V. (2011). Myocardial Revascularization by Coronary Arterial Bypass Graft: Past, Present, and Future. Curr. Probl. Cardiol..

[5] Rocha E.A.V. (2017). Fifty Years of Coronary Artery Bypass Graft Surgery. Braz. J. Cardiovasc. Surg..

[6] Bondarenko O., Knaapen P., van Rossum A.C. (2005). Transient Pericardial Effusion after Cardiac Surgery: Often Unrecognised. Heart.

[7] Liblik K., Zucker J., Baranchuk A., Fernandez A.L., Zhang S., Diasty M.E. (2024). The Role of Pericardial Fluid Biomarkers in Predicting Post-Operative Atrial Fibrillation, a Comprehensive Review of Current Literature. Trends Cardiovasc. Med..

[8] Feng X., Wu F., Wu Y., Ding S., Tao X., Li J., Liu W., Ma R., Chen Y. (2022). A Prediction Rule Including Interleukin-6 in Pericardial Drainage Improves Prediction of New-Onset Atrial Fibrillation After Coronary Artery Bypass Grafting. J. Cardiothorac. Vasc. Anesth..

[9] Liu Y., Wu F., Wu Y., Elliott M., Zhou W., Deng Y., Ren D., Zhao H. (2021). Mechanism of IL-6-Related Spontaneous Atrial Fibrillation after Coronary Artery Grafting Surgery: *IL-6* Knockout Mouse Study and Human Observation. Transl. Res..

[10] Fatehi Hassanabad A., Schoettler F.I., Kent W.D.T., Adams C.A., Holloway D.D., Ali I.S., Novick R.J., Ahsan M.R., McClure R.S., Shanmugam G. (2022). Comprehensive Characterization of the Postoperative Pericardial Inflammatory Response: Potential Implications for Clinical Outcomes. JTCVS Open.

[11] Manghelli J.L., Kelly M.O., Carter D.I., Gauthier J.M., Scozzi D., Lancaster T.S., MacGregor R.M., Khiabani A.J., Schuessler R.B., Gelman A.E. (2021). Pericardial Mitochondrial DNA Levels Are Associated With Atrial Fibrillation After Cardiac Surgery. Ann. Thorac. Surg..

[12] Liu Y., Yu M., Wu Y., Wu F., Feng X., Zhao H. (2023). Myeloperoxidase in the Pericardial Fluid Improves the Performance of Prediction Rules for Postoperative Atrial Fibrillation. J. Thorac. Cardiovasc. Surg..

[13] Liu Y., Yang Y., Yang X., Hua K. (2022). Myeloperoxidase Levels in Pericardial Fluid Is Independently Associated with Postoperative Atrial Fibrillation after Isolated Coronary Artery Bypass Surgery. J. Clin. Med..

[14] Yang Y., Du Z., Fang M., Ma Y., Liu Y., Wang T., Han Z., Peng Z., Pan Y., Qin H. (2023). Metabolic Signatures in Pericardial Fluid and Serum Are Associated with New-Onset Atrial Fibrillation after Isolated Coronary Artery Bypass Grafting. Transl. Res..

[15] Nakamura T., Azuma A., Sawada T., Sakamoto K., Yamano T., Yaku H., Matsubara H. (2007). Brain Natriuretic Peptide Concentration in Pericardial Fluid Is Independently Associated with Atrial Fibrillation after Off-Pump Coronary Artery Bypass Surgery. Coron. Artery Dis..

[16] Fragão-Marques M., Barroso I., Farinha R., Miranda I.M., Martins D., Mancio J., Rocha-Neves J., Guimarães J.T., Leite-Moreira A., Falcão-Pires I. (2021). Pericardial NT-Pro-BNP and GDF-15 as Biomarkers of Atrial Fibrillation and Atrial Matrix Remodeling in Aortic Stenosis. Diagnostics.

[17] Van Boven W.J., de Groot J.R., Kluin J. (2021). A Short Cut to Prevent Postoperative Atrial Fibrillation. Lancet.

[18] van den Berg N.W.E., Neefs J., Kawasaki M., Nariswari F.A., Wesselink R., Fabrizi B., Jongejan A., Klaver M.N., Havenaar H., Hulsman E.L. (2021). Extracellular Matrix Remodeling Precedes Atrial Fibrillation: Results of the PREDICT-AF Trial. Heart Rhythm.

[19] van der Heijden C.A.J., Verheule S., Olsthoorn J.R., Mihl C., Poulina L., van Kuijk S.M.J., Heuts S., Maessen J.G., Bidar E., Maesen B. (2022). Postoperative Atrial Fibrillation and Atrial Epicardial Fat: Is There a Link?. Int. J. Cardiol. Heart Vasc..

[20] Meenashi Sundaram D., Vasavada A.M., Ravindra C., Rengan V., Meenashi Sundaram P. (2023). The Management of Postoperative Atrial Fibrillation (POAF): A Systematic Review. Cureus.

[21] Gaudino M., Di Franco A., Rong L.Q., Piccini J., Mack M. (2023). Postoperative Atrial Fibrillation: From Mechanisms to Treatment. Eur. Heart J..

[22] Greenberg J.W., Lancaster T.S., Schuessler R.B., Melby S.J. (2017). Postoperative Atrial Fibrillation Following Cardiac Surgery: A Persistent Complication. Eur. J. Cardiothorac. Surg..

[23] St-Onge S., Perrault L.P., Demers P., Boyle E.M., Gillinov A.M., Cox J., Melby S. (2018). Pericardial Blood as a Trigger for Postoperative Atrial Fibrillation After Cardiac Surgery. Ann. Thorac. Surg..

[24] Price S., Prout J., Jaggar S.I., Gibson D.G., Pepper J.R. (2004). “Tamponade” Following Cardiac Surgery: Terminology and Echocardiography May Both Mislead. Eur. J. Cardiothorac. Surg..

[25] Kulik A., Rubens F.D., Wells P.S., Kearon C., Mesana T.G., van Berkom J., Lam B.-K. (2006). Early Postoperative Anticoagulation after Mechanical Valve Replacement: A Systematic Review. Ann. Thorac. Surg..

[26] Pepi M., Muratori M., Barbier P., Doria E., Arena V., Berti M., Celeste F., Guazzi M., Tamborini G. (1994). Pericardial Effusion after Cardiac Surgery: Incidence, Site, Size, and Haemodynamic Consequences. Br. Heart J..

[27] Ashikhmina E.A., Schaff H.V., Sinak L.J., Li Z., Dearani J.A., Suri R.M., Park S.J., Orszulak T.A., Sundt T.M. (2010). Pericardial Effusion After Cardiac Surgery: Risk Factors, Patient Profiles, and Contemporary Management. Ann. Thorac. Surg..

[28] Ikäheimo M.J., Huikuri H.V., Airaksinen K.E.J., Korhonen U.-R., Linnaluoto M.K., Tarkka M.R., Takkunen J.T. (1988). Pericardial Effusion after Cardiac Surgery: Incidence, Relation to the Type of Surgery, Antithrombotic Therapy, and Early Coronary Bypass Graft Patency. Am. Heart J..

[29] Al-Dadah A.S., Guthrie T.J., Pasque M.K., Moon M.R., Ewald G.A., Moazami N. (2007). Clinical Course and Predictors of Pericardial Effusion Following Cardiac Transplantation. Transpl. Proc..

[30] Lehto J., Kiviniemi T. (2020). Postpericardiotomy Syndrome after Cardiac Surgery. Ann. Med..

[31] Gabaldo K., Sutlić Ž., Mišković D., Knežević Praveček M., Prvulović Đ., Vujeva B., Cvitkušić Lukenda K., Hadžibegović I. (2019). Postpericardiotomy Syndrome Incidence, Diagnostic and Treatment Strategies: Experience AT Two Collaborative Centers. Acta Clin. Croat..

[32] Imazio M., Brucato A., Rovere M.E., Gandino A., Cemin R., Ferrua S., Maestroni S., Barosi A., Simon C., Ferrazzi P. (2011). Contemporary Features, Risk Factors, and Prognosis of the Post-Pericardiotomy Syndrome. Am. J. Cardiol..

[33] Lehto J., Gunn J., Karjalainen P., Airaksinen J., Kiviniemi T. (2015). Incidence and Risk Factors of Postpericardiotomy Syndrome Requiring Medical Attention: The Finland Postpericardiotomy Syndrome Study. J. Thorac. Cardiovasc. Surg..

[34] Zampieri F.G., Jacob V., Barbeiro H.V., Pinheiro da Silva F., de Souza H.P. (2015). Influence of Body Mass Index on Inflammatory Profile at Admission in Critically Ill Septic Patients. Int. J. Inflam..

[35] Borkon A.M., Schaff H.V., Gardner T.J., Merrill W.H., Brawley R.K., Donahoo J.S., Watkins L., Weiss J.L., Gott V.L. (1981). Diagnosis and Management of Postoperative Pericardial Effusions and Late Cardiac Tamponade Following Open-Heart Surgery. Ann. Thorac. Surg..

[36] Tomić S., Djokić O., Babić S., Raičković T., Mićović S. (2020). The Use of Arterial Grafts of the Left Internal Mammary Artery Is Not a Predictor for the Incidence of Pericardial Effusion. Open Access Maced. J. Med. Sci..

[37] Seese L., Ashraf S.F., Davila A., Coyan G., Joubert K., Zhang D., Kaczorowski D., West D., Sultan I., Bonatti J. (2023). Robotic Totally Endoscopic Coronary Artery Bypass Grafting—Port Placements, Internal Mammary Artery Harvesting and Anastomosis Techniques. J. Vis. Surg..

[38] Smoczyñski R., Staromłyñski J., Bartczak M., Kowalewski M., Pawłowski T., Gil R., Drobiñski D., Król Z., Wierzba W., Suwalski P. (2022). Bilateral Internal Mammary Artery in Coronary Artery Bypass Grafting Using the Latest Da Vinci Xi Robot. Kardiochir. Torakochirurgia Pol..

[39] Mohammed Yusuf M., Bansal V., Venkatesh A., Mahesh Kumar A. (2024). Double Docking Technique for Bilateral Internal Mammary Artery Harvest. https://ctsnet.figshare.com/articles/media/Double_Docking_Technique_for_Bilateral_Internal_Mammary_Artery_Harvest/25360711/1.

[40] Bonatti J. (2024). Robotically Assisted Internal Mammary Artery Harvesting—Will Single-Port Systems Be Useful?. Interdiscip. CardioVascular Thorac. Surg..

[41] Buoro S., Tombetti E., Ceriotti F., Simon C., Cugola D., Seghezzi M., Innocente F., Maestroni S., Del Carmen Baigorria Vaca M., Moioli V. (2021). What Is the Normal Composition of Pericardial Fluid?. Heart.

[42] Anthony S.R., Guarnieri A.R., Gozdiff A., Helsley R.N., Phillip Owens A., Tranter M. (2019). Mechanisms Linking Adipose Tissue Inflammation to Cardiac Hypertrophy and Fibrosis. Clin. Sci..

[43] Azarbal A., LeWinter M.M. (2017). Pericardial Effusion. Cardiol. Clin..

[44] Adler Y., Charron P. (2015). The 2015 ESC Guidelines on the Diagnosis and Management of Pericardial Diseases. Eur. Heart J..

[45] Light R.W., Macgregor M.I., Luchsinger P.C., Ball W.C. (1972). Pleural Effusions: The Diagnostic Separation of Transudates and Exudates. Ann. Intern. Med..

[46] Ben-Horin S., Shinfeld A., Kachel E., Chetrit A., Livneh A. (2005). The Composition of Normal Pericardial Fluid and Its Implications for Diagnosing Pericardial Effusions. Am. J. Med..

[47] Fender E.A., Zack C.J. (2021). Shining a New Light on Pericardial Fluid. Heart.

[48] Imazio M., Biondo A., Ricci D., Boffini M., Pivetta E., Brucato A., Giustetto C., De Ferrari G.M., Rinaldi M. (2020). Contemporary Biochemical Analysis of Normal Pericardial Fluid. Heart.

[49] Bai W., Chen J., Mao Y., Wang Z., Qian X., Hu X., Xu K., Pan Y. (2020). A Predictive Model for the Identification of Cardiac Effusions Misclassified by Light’s Criteria. Lab. Med..

[50] Melduni R.M., Schaff H.V., Bailey K.R., Cha S.S., Ammash N.M., Seward J.B., Gersh B.J. (2015). Implications of New-Onset Atrial Fibrillation after Cardiac Surgery on Long-Term Prognosis: A Community-Based Study. Am. Heart J..

[51] Gaudino M., Di Franco A., Rong L.Q., Cao D., Pivato C.A., Soletti G.J., Chadow D., Cancelli G., Perezgrovas Olaria R., Gillinov M. (2022). Pericardial Effusion Provoking Atrial Fibrillation After Cardiac Surgery: JACC Review Topic of the Week. J. Am. Coll. Cardiol..

[52] Dobrev D., Aguilar M., Heijman J., Guichard J.-B., Nattel S. (2019). Postoperative Atrial Fibrillation: Mechanisms, Manifestations and Management. Nat. Rev. Cardiol..

[53] Gozdek M., Pawliszak W., Hagner W., Zalewski P., Kowalewski J., Paparella D., Carrel T., Anisimowicz L., Kowalewski M. (2017). Systematic Review and Meta-Analysis of Randomized Controlled Trials Assessing Safety and Efficacy of Posterior Pericardial Drainage in Patients Undergoing Heart Surgery. J. Thorac. Cardiovasc. Surg..

[54] Gaudino M., Sanna T., Ballman K.V., Robinson N.B., Hameed I., Audisio K., Rahouma M., Di Franco A., Soletti G.J., Lau C. (2021). Posterior Left Pericardiotomy for the Prevention of Atrial Fibrillation after Cardiac Surgery: An Adaptive, Single-Centre, Single-Blind, Randomised, Controlled Trial. Lancet.

[55] Abouarab A.A., Leonard J.R., Ohmes L.B., Lau C., Rong L.Q., Ivascu N.S., Pryor K.O., Munjal M., Crea F., Massetti M. (2017). Posterior Left Pericardiotomy for the Prevention of Postoperative Atrial Fibrillation after Cardiac Surgery (PALACS): Study Protocol for a Randomized Controlled Trial. Trials.

[56] Kaireviciute D., Blann A.D., Balakrishnan B., Lane D.A., Patel J.V., Uzdavinys G., Norkunas G., Kalinauskas G., Sirvydis V., Aidietis A. (2010). Characterisation and Validity of Inflammatory Biomarkers in the Prediction of Post-Operative Atrial Fibrillation in Coronary Artery Disease Patients. Thromb. Haemost..

[57] Turagam M.K., Mirza M., Werner P.H., Sra J., Kress D.C., Tajik A.J., Jahangir A. (2016). Circulating Biomarkers Predictive of Postoperative Atrial Fibrillation. Cardiol. Rev..

[58] Zhang Q., Raoof M., Chen Y., Sumi Y., Sursal T., Junger W., Brohi K., Itagaki K., Hauser C.J. (2010). Circulating Mitochondrial DAMPs Cause Inflammatory Responses to Injury. Nature.

[59] Heissig B., Nishida C., Tashiro Y., Sato Y., Ishihara M., Ohki M., Gritli I., Rosenkvist J., Hattori K. (2010). Role of Neutrophil-Derived Matrix Metalloproteinase-9 in Tissue Regeneration. Histol. Histopathol..

[60] Wilson W.R.W., Anderton M., Schwalbe E.C., Jones J.L., Furness P.N., Bell P.R.F., Thompson M.M. (2006). Matrix Metalloproteinase-8 and -9 Are Increased at the Site of Abdominal Aortic Aneurysm Rupture. Circulation.

[61] Oka T., Hikoso S., Yamaguchi O., Taneike M., Takeda T., Tamai T., Oyabu J., Murakawa T., Nakayama H., Nishida K. (2012). Mitochondrial DNA That Escapes from Autophagy Causes Inflammation and Heart Failure. Nature.

[62] Sandler N., Kaczmarek E., Itagaki K., Zheng Y., Otterbein L., Khabbaz K., Liu D., Senthilnathan V., Gruen R.L., Hauser C.J. (2018). Mitochondrial DAMPs Are Released During Cardiopulmonary Bypass Surgery and Are Associated With Postoperative Atrial Fibrillation. Heart Lung Circ..

[63] DeBerardinis R.J., Keshari K.R. (2022). Metabolic Analysis as a Driver for Discovery, Diagnosis, and Therapy. Cell.

[64] Würtz P., Havulinna A.S., Soininen P., Tynkkynen T., Prieto-Merino D., Tillin T., Ghorbani A., Artati A., Wang Q., Tiainen M. (2015). Metabolite Profiling and Cardiovascular Event Risk. Circulation.

[65] Floegel A., Kühn T., Sookthai D., Johnson T., Prehn C., Rolle-Kampczyk U., Otto W., Weikert C., Illig T., von Bergen M. (2018). Serum Metabolites and Risk of Myocardial Infarction and Ischemic Stroke: A Targeted Metabolomic Approach in Two German Prospective Cohorts. Eur. J. Epidemiol..

[66] Razquin C., Ruiz-Canela M., Toledo E., Hernández-Alonso P., Clish C.B., Guasch-Ferré M., Li J., Wittenbecher C., Dennis C., Alonso-Gómez A. (2021). Metabolomics of the Tryptophan–Kynurenine Degradation Pathway and Risk of Atrial Fibrillation and Heart Failure: Potential Modification Effect of Mediterranean Diet. Am. J. Clin. Nutr..

[67] Suffee N., Baptista E., Piquereau J., Ponnaiah M., Doisne N., Ichou F., Lhomme M., Pichard C., Galand V., Mougenot N. (2022). Impacts of a High-Fat Diet on the Metabolic Profile and the Phenotype of Atrial Myocardium in Mice. Cardiovasc. Res..

[68] Zuo K., Fang C., Liu Z., Fu Y., Liu Y., Liu L., Wang Y., Yin X., Liu X., Li J. (2022). Commensal Microbe-Derived SCFA Alleviates Atrial Fibrillation via GPR43/NLRP3 Signaling. Int. J. Biol. Sci..

[69] Li X.-Y., Hou H.-T., Chen H.-X., Liu X.-C., Wang J., Yang Q., He G.-W. (2021). Preoperative Plasma Biomarkers Associated with Atrial Fibrillation after Coronary Artery Bypass Surgery. J. Thorac. Cardiovasc. Surg..

[70] Holzwirth E., Kornej J., Erbs S., Obradovic D., Bollmann A., Hindricks G., Thiele H., Büttner P. (2020). Myeloperoxidase in Atrial Fibrillation: Association with Progression, Origin and Influence of Renin–Angiotensin System Antagonists. Clin. Res. Cardiol..

[71] Nicholls S.J., Hazen S.L. (2005). Myeloperoxidase and Cardiovascular Disease. ATVB.

[72] Zhang R., Shen Z., Nauseef W.M., Hazen S.L. (2002). Defects in Leukocyte-Mediated Initiation of Lipid Peroxidation in Plasma as Studied in Myeloperoxidase-Deficient Subjects: Systematic Identification of Multiple Endogenous Diffusible Substrates for Myeloperoxidase in Plasma. Blood.

[73] Zhang R., Brennan M.-L., Shen Z., MacPherson J.C., Schmitt D., Molenda C.E., Hazen S.L. (2002). Myeloperoxidase Functions as a Major Enzymatic Catalyst for Initiation of Lipid Peroxidation at Sites of Inflammation. J. Biol. Chem..

[74] Michaud K., Augsburger M., Donzé N., Sabatasso S., Faouzi M., Bollmann M., Mangin P. (2008). Evaluation of Postmortem Measurement of NT-ProBNP as a Marker for Cardiac Function. Int. J. Leg. Med..

